# Supplementary material for: Mitochondrial genome of eight Carangidae and phylogenetic analysis in the family
Source: PLoS One. 2025 Jul 9;20(7):e0326619. doi: 10.1371/journal.pone.0326619 (PMC12240318; doi:10.1371/journal.pone.0326619)
Supplement: Attachment — Submitted filename: comments.docx [file pone.0326619.s001.docx]

Dear Authors

I hope this message finds you well.

The author has conducted valuable research on the mitochondrial genome assembly of eight Carangidae species and the phylogenetic analysis of the Carangidae family. While the article is well-written overall, there are some suggestions and corrections needed to enhance its quality.

I kindly request your assistance in improving the overall language quality of the article to enhance its clarity and readability. Additionally, I noticed that the figures provided could benefit from quality improvements to better support the content of the manuscript.

Your expertise in refining the text and upgrading the figures would significantly improve the overall presentation of the article. Below, I have outlined the suggestions and corrections for your consideration:

1). Title: The title needs to be revised. It is not well constructed. I suggest directly mentioning mitochondrial genomes and their phylogenetic analysis. The family name appears twice, and the mention of "assembly" is unnecessary.

2) Line 28: Change "Genome sizes were" to "Mitochondrial genome sizes were."

3) Line 33: Clarify the sentence. Did you mean that these genera were sequenced for the first time or these species? If it is the species, why were the others sequenced again?

4) Introduction, Line 46: Revise this sentence for better clarity.

5)Lines 69–72: Provide relevant citations and specify the results. Are these results based on mitochondrial genomes? Clarify which datasets were used and whether the phylogeny focuses on a species, family, or genus.

6) Line 94: Revise this line for clarity.

7) Table 1: Ensure all Latin names are italicized. Review the entire table for consistency. In the legends, change "Genric" to "Genus."

8) Line 157: Add references to support the statement "nucleotide skewness observed in other vertebrate mitochondrial genomes." Specify which studies or examples you are referring to?.

9) Table 2: Explain why you provided GC percentages but not AT content. Alternatively, consider providing both values for completeness.

10) Table 2 Length: The table is too long. Consider presenting data for one or two species in the main table and moving the rest to supplementary materials.

11) Table 3: This table does not look appropriate in the main text. It should be moved to the supporting materials.

12) Line 224: Revise the sentence "Alepes djedaba and A. kleinii clustered in the same genus within Carangidae." Ensure that species names are italicized.

13) Line 279: You mentioned that new classification problems were identified. Clearly explain these issues and highlight them in your results. This will enhance the value of your discovery and the overall article.

14) Figure 1: Revise the caption to read. Add map. Like "Mitochondrial genome maps of eight..." Also, improve the figure's quality.

15) Figures 6 and 7: It seems like these figures are the same, but you mentioned them as ML and BI analyses. Clarify this and confirm the nodal values to avoid confusion. According to my knowledge every BI results values must be 0 to 1 values? but here you use same as ML?.

16) Are your phylogenetic results based on the 13 protein-coding genes (PCGs)? I couldn’t find a clear explanation in the manuscript regarding whether your phylogenetic analysis is based on 13 PCGs, PCG + rRNA datasets, or the whole genome. Please specify this information clearly in the methods section and in the figure captions. For example, indicate in the ML tree figure caption what dataset the analysis is based on. This will make your phylogenetic analysis more transparent and understandable.

17) How many generations did you run for the Bayesian Inference (BI) analysis? Please include the details about the number of generations for BI in the Methods and Materials section for clarity.

18) Why did you use different models for the Maximum Likelihood (ML) and Bayesian Inference (BI) analyses? Typically, consistency in model selection ensures comparability between phylogenetic methods. Please clarify the rationale behind using different models and explain how it impacts the results in your analysis.

19) Conclusion (Line 323): The sentence "This study assembled the mtDNAs of eight Carangidae species (including three for the first time)" needs clarification. Are you referring to the genera or species being sequenced for the first time? What about the remaining species? Revise for better understanding.

Please review them and make the necessary revisions. Once these corrections are made, the manuscript will be in great shape for publication.

Thank you for your time and support.
